# 

**Published:** 1791

**Authors:** 


LONDON:

PRINTED FOR J. JOHNSON, N° 72, ST. PAUL'S CHURCH YARD.

M.DCC.XCI.

DIRECTIONS TO THE BINDER.

Plate the Firſt, the ſubjects of which are deſcribed in pages 100 and 104, may be placed at page 100; and Plate the Second, at page 164.

MEDICAL

### CATALOGUE OF BOOKS.

1. A NEW Tranflation of the Pharmacopœia of the Royal College of Phyficians of London, of the Year 1787; with Notes critical and explanatory; Dofes of the feveral Preparations; likewife a Table of the quantities of Opium and Quickfilver in the compound Medicines which contain them; and a Lift of the new Names, together with Latin and Englifh Indexes. By an Apothecary. 8vo. *Johnfon*, London, 1789.

2. Obfervations on the Duties of a Phyfician, and the Methods of improving Medicine; accomodated to the prefent State of Society and Manners in the United States: delivered in the Univerfity of Pennfylvania on the 7th of February, 1789, at the Conclufion of a Courfe of Lectures upon Chemiftry and the Practice of Phyfic. By *Benjamin Rufh*, M. D. 8vo. Philadelphia, 1789.

3. Practical Hints on Opium confidered as a Poifon. By *R. Hamilton*, M. D. 8vo. Ipfwich, 1790.

4. Thoughts Phyfiological, Pathological, and Practical, with fome Cafes, and Anatomico-practical tical Obfervations. By *Allen Swainfton*, M. D. at York. 8vo. York, 1790.

5. A Letter to Sir John Sinclair, Bart. concerning the Virtues of the Muriatic Acid, or Spirit of Sea Salt, in the Cure of Putrid Difeafes. By Sir *William Fordyce*, M. D. F. R. S. 8vo. *Cadell*, London, 1790.

6. An Effay on Fevers; wherein their theoretic Genera, Species and various Denominations, are, from Obfervation and Experience for thirty Years, in Europe, Africa, and America, and the intermediate Seas, reduced under their Characteriftic Genus, febrile Infection; and the Cure eftablifhed on Philofophical Induction. By *Robert Robertfon*, M. D. a Surgeon of his Majefty's Navy. 8vo. *Robinfons*, London, 1790.

7. An Enquiry into the Small Pox, Medical and Political; wherein a fuccefsful Method of treating that Difeafe is propofed; the Caufe of Pits explained; and the Method of their Prevention pointed out. With an Appendix, reprefenting the prefent State of the Small Pox. By *Robert Walker*, M. D. Fellow of the Royal College of Surgeons, Edinburgh. 8vo. London, 1790.

8. A Treatife on Putrid Inteftinal Remitting Fevers; in which the Laws of the Febrile State,

State, and Sol-Lunar Influence, being investigated and defined, are applied to explain the Nature of the various Forms, Crises, and other Phænomena of these Fevers: and thence is deduced and instituted an improved Method of curing them. By *Francis Balfour*, M. D. Soc. Reg. Med. Ed. S. H. 8vo. Edinburgh, 1790.

9. Elements of Chemistry, in new Systematic Order, containing all the modern Discoveries; illustrated with thirteen Copper Plates. By M. *Lavoisier*, Member of the Academy of Sciences, &c. Translated from the French, by *Robert Kerr*, F. R. & A. S. S. Ed. Member of the Royal College of Surgeons, and Surgeon to the Orphan Hospital, Edinburgh. 8vo. Edinburgh, 1790.

10. Speculations on the Mode and Appearances of Impregnation in the Human Female, with an Examination of the present theories on Generation. By a Physician. 8vo. Edinburgh, 1790.

11. Elements of Natural History and Chemistry. By M. *Fourcroy*, Doctor of the Faculty of Medicine at Paris, &c. Translated from the last Paris Edition, 1789, being the third, in five vols, 8vo. With an Alphabetical comparative View of the ancient and modern modern Names of Chemical Subftances, with all the Tables, and a complete Index. To which is prefixed, by the fame Tranflator, a Preface, containing Strictures on the Hiftory of the prefent State of Chemiftry; with Obfervations on the Pofitions, Facts and Arguments, urged for and againft the Antiphlogiftic Theory and new Nomenclature, by Meffrs. Lavoifier, Prieftley, Kirwan, Keir, Sage, &c. 8vo. 3 Vols. *Elliot* and Co. London, 1790.

12. On the Principle of Vitality; a Difcourfe delivered in the firft Church in Bofton, Tuefday, June 8th 1790, before the Humane Society of the Commonwealth of Maffachufets. By *B. Waterhoufe*, M. D. Profeffor of the Theory and Practice of Phyfic, and Lecturer on Natural Hiftory in the Univerfity of Cambridge. 4to. Bofton, 1790.

13. An Inaugural Differtation * on the Phenomena, caufes and effects of Fermentation; fubmitted to the Provoft, Truftees, and Medical Profeffors of the College of Philadelphia, for

* By a late Regulation of the College of Philadelphia a Candidate for the Degree of Doctor of Phyfic is permitted to write his Thefis either in the Latin or Englifh Language. This is the firft that has been written in Englifh.

the the Degree of Doctor of Medicine; June 1790. By *John Pennington.* 8vo. Philadelphia, 1790.

14. A Treatise on the Inoculation of Horses for the Strangles: in which is clearly laid down the Manner and Time of the Operation, the Preparation necessary previous thereto, and the Mode of Treatment during the continuance of the Disorder: the Whole being the Result of long and repeated Experience. By *Richard Ford,* of Birmingham; who has made the Complaints of Horses his Study, for more than fifty Years past. 12mo. Birmingham, 1790.

15. The Philosophy of Natural History. By *William Smellie,* Member of the Antiquarian and Royal Societies of Edinburgh. 4to. Edinburgh, 1790.

16. The Sexes of Plants vindicated; in a Letter to Mr. William Smellie; containing a refutation of his Arguments against the Sexes of Plants, and Remarks on certain Passages of his Philosophy of Natural History. By *John Rotheram,* M. D. Fellow of the Linnæan Society, London. 8vo. *Cadell,* London, 1790.

17. Observations on Animal Life, and apparent Death, from accidental Suspension of the Function of the Lungs; with Remarks on the

Bruno-

Brunonian Syftem of Medicine. By *J. Franks.* 8vo. *Johnfon*, London, 1790.

18. An Effay on a Non-Defcript, or newly-invented Difeafe; its Nature, Caufes, and Means of Relief: With fome very important Obfervations on the powerful and moft furprifing Effects of Animal Magnetifm in the Cure of the faid Difeafe, as communicated to the Author by Dr. Mefmer and Madame de L—g; and a Dedication to the faid Lady. By *F. G.*, Profeffor of Phyfic and Aftrology, and Member of feveral learned Academies and Societies. 8vo. *Bateman*, London, 1790.

19. Effays on fafhionable Difeafes; the dangerous Effects of hot and crowded Rooms; the Cloathing of Invalids; Lady and Gentlemen Doctors; and on Quacks and Quackery: With the genuine Patent Prefcriptions of Dr. James's Fever Powder, Tickell's Ætherial Spirit, and Godbold's Balfam, taken from the Rolls in Chancery, and under the Seal of the proper Officers; and alfo the Ingredients and Compofition of many of the moft celebrated Quack Noftrums, as analyfed by feveral of the beft Chemifts in Europe. By *James M. Adair*, formerly M. D., Member of the Royal Medical Society, Fellow of the Royal College of Phyficians ficians of Edinburgh; Phyfician to the Commander in Chief of the Leeward Iflands, and to the Colonial Troops; and one of the Judges of the Courts of King's Bench and Common Pleas in the Ifland of Antigua. 8vo. *Bateman*, London, 1790.

20. A candid Inquiry into the Truth of certain Charges of the dangerous Confequences of the Suttonian, or cooling Regimen, under Inoculation for the Small Pox. Recommended to the ferious Confideration of Parents and Guardians, as being of the utmoft Importance to the Welfare of the rifing Generation. With fome ufeful Remarks on a fuccefsful Method, ufed fome Years ago in Hungary, in the Cure of the natural Small Pox, and tending to demonftrate the Benefit to be expected from a fimilar Method of Management under Inoculation. By *James M'. Adair*, formerly M. D. &c. 8vo. *Bateman*, London, 1790.

21. A Treatife on Air, containing new Experiments and Thoughts on Combuftion; being a full Inveftigation of Mr. Lavoifier's Syftem, and proving, by fome ftriking Experiments, its erroneous Principles; with Strictures upon the chemical Opinions of fome eminent nent Men. By *Richard Bewley*, M. D. 8vo. *Evans*, London, 1790.

22. A Treatiſe of the Plague; containing an hiſtorical Journal and medical Account of the Plague at Aleppo in the Years 1760, 1761, and 1762. Alſo Remarks on Quarantines, Lazarettos, and the Adminiſtration of Police in Times of Peſtilence. To which is added an Appendix, containing Caſes of the Plague, and an Account of the Weather during the peſtilential Seaſon. By *Patrick Ruſſell*, M. D. F. R. S. formerly Phyſician to the Britiſh Factory at Aleppo. 4to. *Robinſons*, London, 1791.

23. Experiments and Obſervations on the Anguſtura Bark. By *Auguſtus Everard Brande*, Apothecary to the Queen. 8vo. *Payne*, London, 1791.

24. A Treatiſe on the Fevers of Jamaica; with ſome Obſervations on the Intermittent Fever of America: and an Appendix, containing ſome Hints on the Means of preſerving the Health of Soldiers in hot Climates. By *Robert Jackſon*, M. D. 8vo. *Murray*, London, 1791.

25. A Treatiſe on the Extraction of the Cataract.

taract. By *D. Augustus Gottlieb Richter*, M. and Ch. D. Aulic Counsellor and Physician to His Britannic Majesty, Professor of the Practice of Physic and Surgery in the University of Gottingen, President of the College of Surgeons, and Member of the Royal Academies of Gottingen, Stockholm, and Copenhagen. Translated from the German, with a Plate, and Notes by the Translator. 8vo. *Murray*, London, 1791.

26. Dissertatio Medica Inauguralis de Hysteria; Auctore *Carolo Bankhead*, Hiberno. 8vo. Edinburgi, 1790.

27. Dissertatio Medica Inauguralis de Febre Typhoidea; Auctore *Henrico Bowles*, Anglo. 8vo. Edin. 1790.

28. Tentamen Medicum Inaugurale de Teretibus Intestinorum Lumbricis; Auctore *Carolo Daly*, Hiberno. 8vo. Edin. 1790.

29. Dissertatio Medica Inauguralis de Asthmate; Auctore *Gulielmo Dick*, Hiberno. 8vo. Edin. 1790.

30. Dissertatio Inauguralis complectens Observationes quasdam de Febre; Auctore *Thoma Evans*, Hiberno. 8vo. Edin. 1790.

31. Dissertatio Medica Inauguralis de Menorrhagia; Auctore *Richardo Feild*, Virginiense. 8vo. Edin. 1790.

32. Tentamen Medicum Inaugurale de Inflammatione; Auctore *Johanne Gahagan*, Hiberno. 8vo. Edin. 1790.

33. Dissertatio Chemica Inauguralis de Æthere; Auctore *Joanne Gibney*, Hiberno. 8vo. Edin. 1790.

34. Dissertatio Medica Inauguralis de Gonorrhœa virulenta; Auctore *Gulielmo Gibson*, Scoto-Britanno. 8vo. Edin. 1790.

35. Tentamen Medicum Inaugurale de Gastritide; Auctore *Jacobo Johnston*, Hiberno. 8vo. Edin. 1790.

36. Dissertatio Medica Inauguralis quædam de Scorbuto, Typho, Variola et Podagra proponens; Auctore *Martino Lynch*, Hiberno. 8vo. Edin. 1790.

37. Dissertatio Medica Inauguralis de Erysipelate; Auctore *Joanne M'Cully*, Hiberno. 8vo. Edin. 1790.

38. Dissertatio Medica Inauguralis de Mercurio; Auctore *Roberto Mackintosh*, ex Comitatu Morariensi. 8vo. Edin. 1790.

39. Dissertatio Chemica Inauguralis de Aquis Mineralibus; Auctore *Gulielmo Meade*, Hiberno. 8vo. Edin. 1790.

40. Dissertatio Medica Inauguralis de Podagra; gra; Auctore *Carolo Scott*, Anglo. 8vo. Edin. 1790.

41. Dissertatio Medica Inauguralis de Vasis absorbentibus; Auctore *Georgio Spence*, Jamaicensi. 8vo. Edin. 1790.

42. Dissertatio Medica Inauguralis de Cynanche Inflammatoria; Auctore *Annesley Strean*, Hiberno. 8vo. Edin. 1790.

43. Dissertatio Medica Inauguralis de Pertussi; Auctore *Roberto Wood*, Scoto. 8vo. Edin. 1790.

44. De Usu Glandularum super renalium; nec non de Origine Adipis: Disquisitio Anatomico-philosophica; Auctore *Nicolao D. Riegels*. 4to. Hafniæ, 1790.

45. *C. G. Th. Kortum*, Medici Tremoniensis, Commentarius de vitio Scrofuloso quique inde pendent Morbis secundariis, qui nuper ill. Societatis Reg. Medicorum, quæ Parisiis est, plausum tulit. Tom. II. 8vo. Lemgo, 1790.

46. Medicinæ omnis ævi fata Tabulis exposuit D. *Aug. Fr. Hecker*. Programma cum munus Professoris Medicinæ ordinarii in perantiqua Erfordiensi Academia adiret. 4to. Erfurt, 1790.

47. De Templis Æsculapii Græcis quædam commencommentatus est *Frid. Wilh. Gerke*, Med. Stud. 8vo. Lipsiæ, 1790.

48. *Sam. Gottl. Vogel*, M. D. Consil. Aulic. Prax. Clin. in Univ. Rostoch. Prof. ord. Manuale Praxeos medicæ, Medicorum illum auspicaturorum usui dicatum. Ex editione Germanica recentissima una cum additamentis Auctoris omnibus loco suo suppletis in linguam transtulit Latinam notasque hinc inde adjecit *Joann. Bernard. Keup*, Med. Doctor. Tom. I. 8vo. Stendal, 1790.

49. Dissertatio de Renum Calculo ejusdemque cum aliis Morbis similitudine; Auctore *Ant. Maria. Cova*, M. D. 8vo. Pavia, 1790.

50. Flora Cochinchinensis: sistens plantas in Regno Cochinchina nascentes; quibus accedunt aliæ observatæ in Sinensi Imperio, Africa Orientali, Indiæque locis variis, omnes dispositæ secundum Systema sexuale Linnæanum, labore ac studio *Joannis de Loureiro*, Regiæ Scientiarum Academiæ Ulyssiponensis Socii: olim in Cochinchinâ Catholicæ Fidei Præconis: ibique rebus Mathematicis ac physicis in Aulâ Præfecti. Jussu Acad. R. Scient. in lucem edita. Tom. II. 4to. Ulyssipone, 1790.

51. Fauna Etrusca sistens Insecta, quæ in Provinciis Florentina et Pisana præsertim collegit legit *P. Rossius.* Tom. II. 4to. Livorno, 1790. c. Tab. æneis x.

52. Supplementum Plantarum selectarum, quarum Imagines manu artificiosa doctaque pinxit *Georgius Dionysius Ehret*; occasione haud vulgari in usum publicum collegit D. *Christ. Jacob Trew*; publicavit et illustravit D. *Bened. Christ. Vogel:* in æs incidendas et coloribus vivis ornandas curavit sumtusque fecit *Joh. Elias Haid*, Chalcographus Augustanus. Augsburg, 1790.

53. Analyses Florum e diversis Plantarum generibus, omnes etiam minutissimas eorum externas partes demonstrantes, ad eruendum harum partium characterem genericum, philosophiam botanicam, et generum intimiores affinitates a Natura statutas; Auctore *A. J. G. C. Batch.* 4to. Vol. I. Fascic. i. Tab. I.—x. Fascic. ii. Tab. xi.—xx. Halæ Magdeburgicæ, 1790.

54. Fungi Mecklenburgenses selecti; Auctore *H. J. Tode.* Fascic. I. nova fungorum genera complectens c. Tab. æneis vii. 4to. Luneburg, 1790.

55. Dissertatio Inauguralis Medica de Thermis Marchio-Badensibus; Auctore *Carolo Fri-*

*derico Haug*, Bada-Badensi. 8vo. Argentorati, 1790.

56. Specimen Physicæ generalis sive de Concretione Corporum et Dissolutione; Auctore *Antonio Bucci* in Faventino Gymnasio Philosophiæ Professore. 8vo. Faenza, 1790.

57. *Ant. Josephi Cavanilles* Icones et Descriptiones Plantarum, quæ aut sponte in Hispania crescunt, aut in Hortis hospitantur. Vol. I. Folio. Matriti, 1790.

58. Dissertatio Inauguralis Medico-Therapeutica de Cortice Angusturæ; Auctore *Frid. Alb. Ant. Meyer*, Hamburgense. 8vo, Gottingæ, 1790.

In this dissertation the author gives a botanical description of the *Magnolia Glauca* Linn., on a supposition that the Cortex Angusturæ, as it is called, is the bark of that tree; but, on comparing the Angustura bark with the bark of the Magnolia Glauca, we do not find the least ground for this conjecture*, which, it seems, originated

* Mr. Brande, in an ingenious and valuable work on this subject lately published, (see article 23 of the present Catalogue) observes also, that having procured and dried the barks of two species of *Magnolia*, the *Glauca* and *Grandiflora*, he found them to differ completely from the Angustura.

originated with Mr. Heyer, in a paper on this ſubject inſerted in the Brunſwick Magazine, Part V. for the year 1790.

59. Diſſertatio Inauguralis medica de Cortice Anguſturæ ejuſque uſu medico; Auctore *Franciſco Erneſto Filter*, Nordhuſano. 4to. Jenæ, 1791.

In this diſſertation alſo we find the author mentioning the conjecture lately thrown out in Germany relative to this bark being the produce of the Magnolia Glauca. He relates ſome inſtances of its efficacy in intermittents; and recommends it likewiſe as a tonic and antiſeptic remedy.

60. De Ophthalmia recens natorum; Auctore *Johanne G. Götz*. 4to. Jena, 1791.

61. De nonnullis quæ ad uſum medicum ſuccorum Vegetabilium recentium ſpectant; Auctore *Wilh. Aug. G. Manniſke*. 4to. Jena, 1791.

62. Hiſtoria Chirurgico-anatomica Facultatis Medicæ Ingoldſtadienſis ab Univerſitate anno

Some perſons in this country have aſſerted that the Cortex Anguſturæ is the bark of the *Brucea Antidyſenterica*, or Wooginos of the Abyſſinians; but the dried bark of this ſhrub is found to be very different from the Cortex Anguſturæ.—EDITOR.

1472 condita ad annum 1788; Auctore *Hen. Palmaz Von Leveling*. 4to. Ingoldstadt. 1791.

63. Icones Plantarum Syriæ rariorum, Descriptionibus et Observationibus illustratæ; Auctore *Jacobo Juliano La Billardiere*. Decas prima. 4to. Lutetiæ Parisiorum, 1791.

64. Dissertatio Inauguralis Medica sistens quædam Medicamenta Rossorum Domestica; Auctore *Joanne Friderico Grahl*, Kioviano. 4to. Jenæ, 1790.

We have here an account of the good effects of an infusion of the *Myrica Gale* LINN. (in the proportion of half an ounce of the dried plant to a pint of water) in chronic rheumatism; together with some observations on the medicinal effects of the Russian liquor called *Kwas*.

65. *Josephi Eyerel* Commentaria in *Maximiliani Stollii* Aphorismos de cognoscendis et curandis Febribus. Tom. III. 8vo. Viennæ, 1790.

66. Diss. Inaug. Medico-Botanica de necessitate et utilitate Studii Botanici; Auctore *Ernesto Carolo Rodschied*, Hanoviensi. 8vo. Marburgi, 1790.

INDEX.

### INDEX.

### A.

ABSORBENT Veſſels, Diſſertation on, — 211

Adair, Dr. James M. Eſſays on faſhionable Diſeaſes, 206

———— on the Inoculation of the Small Pox, — — — 207

Æſculapius, Greek Temples of, Work relative to, 211

Æther, Diſſertation on, — — 210

Air, Treatiſe on, — — 207

Anguſtura Bark, Works relative to, 208, 214, 215

———— is neither a ſpecies of Magnolia, nor the Brucea Antidyſenterica, as hath been ſuppoſed, 214

Animal Magnetiſm, Work relative to, — 206

Aſthma, Diſſertation on, — — 209

### B.

Balfour, Dr. F. Treatiſe on Fevers, — 202

Bankhead, Carolus, de Hyſteria, — 209

Batch, A. J. G. C. Analyſes Florum, — 213

Baynham, W. Caſe of an extra-uterine Conception, 73

Bewley, Dr. Richard, Treatiſe on Air, — 207

Billardiere, Jac. Jul. la, Icones Plantarum Syriæ, 216

Bladder, urinary, Caſe in which a Catheter was left in it, and afterwards extracted, — 96

Blagden, R. B. Facts relative to Pemphigus, - 105

Blane, Dr. Gilbert, Account of the Nardus Indica, or Spikenard, — — 153

Body, human, Account of a Change to which it is ſubject, under certain Circumſtances, after Death, - 186

Bowles, Henr. De febre Typhoidea, — 209

Brande, Aug. Everard, Obſ. on the Anguſtura Bark, 208

Bucci, Ant. de Concretione Corporum et Diſſolutione, 214

Burial Grounds, Reaſon why the Soil of them does not perceptibly accumulate, — — 200

Carminative

### C.

Carminative Medicines, Caution with refpect to, — 96
Cataract, Cafes of the Extraction of, 43, 50, 55, 61
———, Extraction of, a frequent Caufe of its Failure pointed out, — — 62
———————, Remarks relative to, — 67
———————, Treatife on, — 208
Catheter, Inftance of one left in the Bladder, and afterwards extracted by an Operation fimilar to that of Lithotomy, 96
———, flexible, Cafe in which one was kept in the Oefophagus during a Month, — 176
Cavanilles, Ant. Jof. de Plantis Hifpanicis, — 214
Cauftics, Obfervations relative to, in the Prevention of Hydrophobia, — — 14
Child, Account of one with a double Head, — 164
Chin Cough, Differtation on, — 211
Conception, extra-uterine, Cafe of, — 73
Cova, Ant. M. de Renum Calculo, — 212
Cullen, Dr. Remarks on his Arrangement of Difeafes, 95
Cully, Johannes Mac, de Eryfipelate, — 210

### D.

Daly, Carolus, de Teretibus Inteftinorum Lumbricis, 209
Denman, Dr. Thomas, his Account of the fpontaneous Evolution of the Fœtus referred to, — 76
———————, Account of a new Fact relative to Menftruation, — — 108
Dick, Gulielmus, de Afthmate, — 209
Dropfy of the Brain, Obfervations on the Treatment and Caufes of, — — 111
———————, Inftance of its originating in Inflammation, — — 125
———————, faid to arife moft frequently from glandular Obftruction and either local or general Plenitude, — — — 126, 129

### E.

Epiglottis, Account of an Inflammation of, — 40
Eryfipelas, Differtation on, — — 210

Evans,

Evans, Thomas, de Febre, — — 209
Excifion, recommended in the Prevention of Hydrophobia, 13
Eyerel, Jofephus, Commentaria in Max. Stollii Aphorifmos de Febribus, —— —— 216

F.

Fauna Etrufca, —— —— 212
Feild, Richardus, de Menorrhagia, —— 209
Fermentation, Differtation on, ——— 204
Ferriar, Dr. John, Cafe of Hydrophobia, —— 1
Ferris, Dr. Samuel, Cafe of *Petechiæ fine Febre*, 79
Fevers, Differtation on, — — 209
——, Effay on, —— —— 202
——, putrid, inteftinal, remitting, Treatife on, 203
—— of Jamaica, Treatife on, ——— 208
Filter, Fran. Erneft. de Cortice Angufturæ, - 215
Flora Cochinchinenfis, — — 212
Fœtus, fpontaneous Evolution of, Cafe of, —— 76
Ford, Edward, Cafe of a Catheter left in the Bladder of a female Patient in drawing off the Urine, —— 96
———————— an imperforate Rectum, 102
——, R. on the Inoculation of Horfes for the Strangles, 205
Fordyce, Sir W. on the Virtues of muriatic Acid, 202
Fourcroy, M. Elements of Nat. Hiftory and Chemiftry, 203
Franks, J. Obf. on animal Life and apparent Death, 205
Fungi Mecklenburgenfes, — — 213

G.

Gahagan, Johannes, de Inflammatione, —— 210

Garcias ab Horto, his Account of the Tabafheer, 149

———————— Figure of the Nardus Indica mentioned, ——— ——— 160

Gaftritis, Differtation on, ——— 210

Generation, Speculations relative to, —— 203

Gibney, Joannes, de Æthere, —— 210

Gibfon, Gulielmus, de Gonorrhœa, —— *ibid.*

Glands, fuper renal, Differtation on, — 211

Goetz, Joh. G. de Ophthalmia recens natorum, 215

Gonorrhœa, Differtation on, — — 210

Gout,

Gout, Differtation on, —— —— 210

Graff, Dr. his Differtation on *Petechiæ fine Febre* referred to, ——— ——— 80

Grahl, J. F. de quibufdam Medicamentis Rofforum domefticis, — —— — 216

Graves, Dr. Robert, Cafe of Meteorifmus Ventriculi, 90

H.

Haid, J. E. Supplementum Plantarum felectarum, 213
Hamilton, Dr. Robert, practical Hints on Opium, 201
Haug, C. F. de Thermis Marchio-Badenfibus, 213
Haygarth, Dr. his Mode of preventing Hydrophobia, 16
Head, double, Account of a Child with, —— 164
Hecker, Aug. F. Medicinæ omnis ævi fata, — 211
Holy Innocents, Burial Ground, fo called, at Paris, Obfervations relative to, — — 186
Home, E. Account of a Child with a double Head, 164
Hydrocephalus internus.—See Dropfy of the Brain.
Hydrophobia, Cafe of, —— —— 1
——————, Appearances on Diffection in, 7, 10
——————, Remarks on the Prevention and Treatment of, 11, 27
—————————— of, by Excifion, 13
—————————— Cauftic, 14
—————————— wafhing the Wound with pure Water, —— 16
—————————— wafhing the Wound with a dilute Solution of lunar Cauftic in Water, —— —— —— 17
Hyfteria, Differtation on, — — 209

I.

Jackfon, Dr. R. Treatife on the Fevers of Jamaica, 208
Jamaica, Treatife on the Fevers of, —— *ibid.*
Imperforate Rectum, Cafe of, —— 102
Inflammation, Differtation on, —— 210
Influenza, its Fatality, in Virginia, mentioned, 73
Ingolftadt, Work relative to the Hiftory of Phyfic there, 215
Johnfton, Jacobus, de Gaftritide, —— 210

Kerr,

K.

Kerr, Robert, Tranfl. of M. Lavoifier's Elements of Chemiftry, —— —— 203
Kidnies, Calculus of, Differtation on, — 212
Kortum, C. G. T. de Vitio Scrophulofo, —— 211

L.

Lavoifier, M. Elements of Chemiftry, —— 203
Leveling, H. Palmaz von, Hift. Chir. Anat. Facultatis Medicæ Ingoldftadienfis, —— — 215
Loftie, W. on the Prevention and Treatment of Hydrophobia, —— —— — 11
Loureiro, Joannes de, Flora Cochinchinenfis, 212
Lynch, Martinus, de Scorbuto, Typho, Variola et Podagra, —— —— 210

M.

Mackintofh, Robertus, de Mercurio, —— 210

Mainwaring, T. Cafe of Inflammation of the Epiglottis, 40

Mannifke, Wilh. A. G. de ufu med. Succorum Vegetabilium, — — — 215

Manoury, M. Cafe of a Gun-fhot Wound of the Mouth, 176

Meade, Gulielmus, de Aquis mineralibus, — 210

Mederer, Profeffor, his Method for the Prevention of Hydrophobia, —— —— 17, 30

Membrane, difcharged by fome Women during Menftruation, defcribed, —— — 104

————, faid to refemble the *decidua*, —— *ibid.*

————, is accompanied with Pain, —— *ibid.*

————, Mode of Treatment recommended in fuch Cafes, —— — — 110

Menorrhagia, Differtation on, —— 209

Menftruation, Fact relative to it not hitherto defcribed, 108

Mercury, its Efficacy in Dropfy of the Brain extolled, 111

———, Inftances of large Dofes of, in a Cafe of Hydrocephalus, —— —— 117

———, Differtation on, —— — 210

Meteorifmus Ventriculi, Inftance of, —— 90

————————, defcribed by M. Sauvages, 93

————————, confidered by Dr. Cullen as a Species of Tympanites, —— —— 95

Meyer, Frid. Alb. Ant. de Cortice Anguftur æ, 214

Monftrous

Monſtrous Child.—See Child.

Mouth, Caſe of a Gun-ſhot Wound of, — 176

Myrica Gale, recommended in chronic Rheumatiſm, 216

N.

Nardus Indica, Account of, — 153
———, aſcertained to be a Species of Andropogon, — — — 158
———, how employed, medicinally, by the Natives of India, — — — — 157
———, highly valued as an Article of Luxury as well as Medicine by the Ancients, — 161, 162
———, an Ingredient in all the ancient Antidotes, — — — — 162
———, Diſeaſes in which it was employed by the Ancients, — — — 163

O.

Ophthalmia of new-born Infants, Work relative to, 215
Opium, practical Hints on, by Dr. R. Hamilton, 201

P.

Pemphigus, Facts relative to, — 105

Pennington, J. Diſſ. on the Phenomena of Fermentation, 204

Percival, Dr. Thomas, on the Dropſy of the Brain, 111

———, Remarks on his Theory of Hydrophobia, — — — — 36

Petechiæ, without Fever, Caſe of, — 79

———, a Diſeaſe ſo named by Dr. Graff, 80

——— alluded to by Riverius, *ib.*

———, how defined by Dr. Duncan. 81

——— Dr. Adair, *ib.*

———, Remarks on, — 88

Pharmacopœia of the College of Phyſicians of London, new Tranſlation of, — — 201

Philadelphia, Regulation of the College of, permitting Theſes to be written in Latin or Engliſh, — 204

Phyſicians, Obſ. on the Duties of, by Dr. Ruſh, 201

Plague, Treatiſe on, — — 208

Q.

Quin, Dr. Remarks on his Work on Hydrocephalus, 132

### R.

Rabies Canina.—See Hydrophobia.

Rectum, imperforate, Case of, — 102

Rheumatism, chronic, Remedy for, — 216

Richter, D. A. G. on the Extraction of the Cataract, 209

Riegels, Nicol. D. de Glandulis super renalibus, nec non de Adipe, — — 211

Robertson, Dr. Robert, Essay on Fevers, — 202

Rodschied, Ern. Carol. de utilitate Studii Botanici, 216

Roncalli, a Liniment recommended by, Effects of, 134

Rossius, P. Fauna Etrusca, — — 212

Rotheram, Dr. John, the Sexes of Plants vindicated, 205

Rumphius, his Account of the Tabasheer, — 148

Rush, Dr. Benj. his Sentiments relative to the Treatment of Hydrophobia, — — 35

————, Obs. on the Duties of a Physician, 201

Russell, Dr. Patrick, his Account of the Tabasheer, 141

————, Treatise of the Plague, — 208

### S.

Sabatier, M. Instances of the Effects of Cauterisation in the Prevention of Hydrophobia, — 14

Sauvages, M. his Account of the Meteorismus Ventriculi referred to, — — 93

Scott, Carolus, de Podagra, — — 210

Scrophula, Work relative to, — 211

Scrophulous Tumours, Liniment for, recommended by Roncalli, — — 134

Simmons, Richard, Case of spontaneous Evolution of the Fœtus, — — 76

Small Pox, Inquiry into, — 202

Smellie, W. Philosophy of Natural History, - 205

————, Remarks on, by Dr. Rotheram, — — *ibid.*

Sore Throat, inflammatory, Dissertation on, — 211

Spain, Plants of, Work relative to, — 214

Sparrow, Richard, of the Extraction of the Cataract, 43

Speculum Oculi, Objections to its Use in the Extraction of the Cataract, — — 65

Spence, Georgius, de Vasis absorbentibus, — 211

Spermaceti, a Substance resembling it, said to have been extracted from the Brain of different Animals, 194

Spikenard —See Nardus.

Staphyloma, Remarks relative to, —— 68

Stoll, Maxim. Aphorifm. de Febribus, — 216

Stomach.—See *Meteorifmus.*

Strean, Anneſley, de Cynanche Inflammatoria, 211

Streitt, Henry, his Account of a Liniment for ſcrophulous Tumours, —— —— 134

Swainſton, Dr. Allen, Thoughts phyſiological, pathological, and practical, —— —— 201

Syria, rare Plants of, Work relative to, —— 216

T.

Tabaſheer, Accounts of, —— —— 141

———, Error of the old Tranſlators of the Arabian Writers concerning it, —— —— 142

———, is a Production of the Arundo Bambos, *Linn.* 143

———, two Sorts of, one genuine, the other factitious, 148, 153

———, Obſ. relative to it, from a Perſian Work, 153

Thouret, M. Account of a Change to which the human Body, under certain Circumſtances, is ſubject after Death, — — — — 186

Tode, H. J. Fungi Mecklenburgenſes, —— 213

Trew, C. J. Supplementum Plantarum Selectarum, 213

Tumours, ſcrophulous, Account of a Liniment for, 134

Typhus, Diſſertation on, —— —— 209

V.

Vogel, B. C. Supplementum Plantarum Selectarum, 213

———, S. Gottl. Manuale Praxeos medicæ, — 212

W.

Walker, Robert, Inquiry into the Small Pox, 202

Waterhouſe, Dr. B. on the Principle of Vitality, 204

Waters, mineral, Works relative to, — 210, 213

Wenzel, Baron de, his Work on the Cataract recommended, —— —— 72

Williams, Mr. J. L. his Account of the Tabaſheer, 152

Wood, Robertus, de Pertuſſi, —— 211

Worms, round, of the Inteſtines, Diſſertation on, 209

Wound, Gun-ſhot, in the Mouth, Caſe of, —— 176

END OF THE FIRST VOLUME.

